# Poly-L-ornithine enhances migration of neural stem/progenitor cells via promoting α-Actinin 4 binding to actin filaments

**DOI:** 10.1038/srep37681

**Published:** 2016-11-22

**Authors:** Hongfei Ge, Anyong Yu, Jingyu Chen, Jichao Yuan, Yi Yin, Wangsheng Duanmu, Liang Tan, Yang Yang, Chuan Lan, Weixiang Chen, Hua Feng, Rong Hu

**Affiliations:** 1 Department of Neurosurgery and Key Laboratory of Neurotrauma, Southwest Hospital, Third Military Medical University, Chongqing 400038, China; 2 Department of Emergency, The First Affiliated Hospital of Zunyi Medical College, Guizhou 563003, China

## Abstract

The recruitment of neural stem/progenitor cells (NSPCs) for brain restoration after injury is a promising regenerative therapeutic strategy. This strategy involves enhancing proliferation, migration and neuronal differentation of NSPCs. To date, the lack of biomaterials, which facilitate these processes to enhance neural regeneration, is an obstacle for the cell replacement therapies. Our previous study has shown that NSPCs grown on poly-L-ornithine (PO) could proliferate more vigorously and differentiate into more neurons than that on Poly-L-Lysine (PLL) and Fibronectin (FN). Here, we demonstrate that PO could promote migration of NSPCs *in vitro*, and the underlying mechanism is PO activates α-Actinins 4 (ACTN4), which is firstly certified to be expessed in NSPCs, to promote filopodia formation and therefore enhances NSPCs migration. Taken together, PO might serve as a better candidate for transplanted biomaterials in the regenerative therapeutic strategy, compared with PLL and FN.

Neural stem/progenitor cells (NSPCs), bearing the potential of proliferation and differentiation into all neural lineage cell types^1^,^2^,^3^, especially homing to the lesions restoring damaged neurovascular structure, have aroused great enthusiasm of investigators to keep an eye on their advantages of regenerating various neurological diseases and injuries, meanwhile, provide a feasible cell source for transplantation therapies^4^. To date, there are two culture systems to collect NSPCs, known as neurosphere culture system and adherent culture system coated with different substrates to mimic the physiological microenvironment. Meanwhile, NSPCs might exhibit different characteristics among various substrates they grown on.

Different substrates hold disparate effects on proliferation, migration and differentiation of NSPCs. Several studies have illustrated that NSPCs, cultured on different substrates, exhibit different proliferation and differentiation potential^5^,^6^,^7^,^8^. We and our colleagues have previously indicated that NSPCs grown on poly-L-ornithine (PO) could proliferate more vigorously and differentiate into more neurons than that on Poly-L-Lysine (PLL) and Fibronectin (FN)^9^. Furthermore, study has shown that NSPCs proliferate in the Subventricular Zone (SVZ), but only a few proliferated NSPCs could migrate to the lesions after ischemic stroke^10^. It implies that the shortage of functional NSPCs in lesions might due to the inhibition of NSPCs migration potential after brain injury. Therefore, how to promote NSPCs migration to the lesions is another significant issue for the use of NSPCs in cell-based therapies. Hence, it is necessary to explore the influence of migration potential of NSPCs on various substrates and its underlying mechanism(s) to look for a better biomaterial for cell replacement therapies.

The isoforms of α-Actinins, approximately 100 kDa and widely expressed in various mammalian cells, interact with actin filaments to regulate cell motility^11^. To date, there are four human α-actinin genes: actinin 1, 2, 3 and 4^12^. Meanwhile, their encoded proteins involve in the organization of cell cytoskeleton to regulate the motility of muscle and non-muscle cells *via* interacting with various cytoskeletal and membrane-associated proteins and crosslinking actin filaments^13^. Especially, α-Actinins 4 (ACTN4), expressed in non-muscle cells, could enhance cancer cell migration and invasion. Previous studies have shown that ACTN4 promotes metastatic potential in numerous malignant cancers cells, including cervical cancer^14^, human pancreatic cancer^15^ and non-small cell lung cancer^16^, by directly linking the actin filaments^17^ and/or indirectly driving the actin filaments to focal adhesion sites through the cross-link with adhesion plaque proteins^18^. Whether the NSPCs, one type of non-muscle cells, express ACTN4 is still unclear. Meanwhile, the role of ACTN4 playing in regulating NSPCs migration on different substrates needs to be further elucidated.

In this present study, three substrates (PO, PLL and FN) were tested for their effects on migration of NSPCs. Meanwhile, the expression of ACTN4 in NSPCs was investigated and its role in regulating actin filaments formation was explored. The aim of this study is to investigate the different effects of the three widely used substrates on the migration of NSPCs, therefore, look for a more suitable biomaterial candidate to mimic the physiological microenvironment for NSPCs transplantation strategies. Furthermore, try to elucidate the possible underlying mechanism(s), which might provide more intervention targets for basic and preclinical research to promote functional recovery after various neurological diseases and injuries.

## Results

### Characteristics of NSPCs isolated from rats

**Figure 1:**
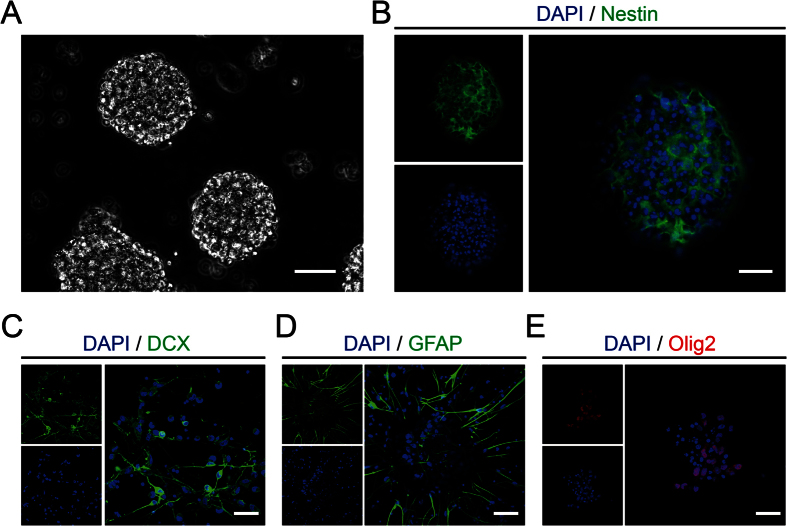
Characteristics and differentiation potential of NSPCs isolated from rats. **(A)** The suspended growth of neurospheres was notably observed after 3 days. **(B)** The immunostaining showed the Nestin expression (green) on NSPCs in neurospheres before seeded on substrates. **(C–E)** The immunostaining demonstrated the differentiation potential of NSPCs into neurons (DCX, green), astrocytes (GFAP, green), or oligodendrocytes (Olig2, red). Cell nuclei were stained with DAPI in blue. Scale bar: 20 μm.

NSPCs, were obtained from neocortical tissues from E14.5 Sprague–Dawley rats as previously described^9^. They were cultivated in enrichment medium (20 ng/ml FGF-basic and 20 ng/ml EGF) and the suspended growth of neurospheres were notably observed after 3 days (Fig. 1A). Meanwhile, immunofluorescence identified cells expressing Nestin (Fig. 1B). In order to test the differentiation potential, the cells from neurospheres were seeded on PO feeded with conditioned medium (without 20 ng/ml FGF-basic and 20 ng/ml EGF) for 10 days. The immunofluorescence results demonstrated that the cells holded the potential to differentiate into neurons (Fig. 1C), astrocytes (Fig. 1D) and oligodendrocytes (Fig. 1E).

### PO evidently enhanced migration of NSPCs by promoting filopodia formation

**Figure 2:**
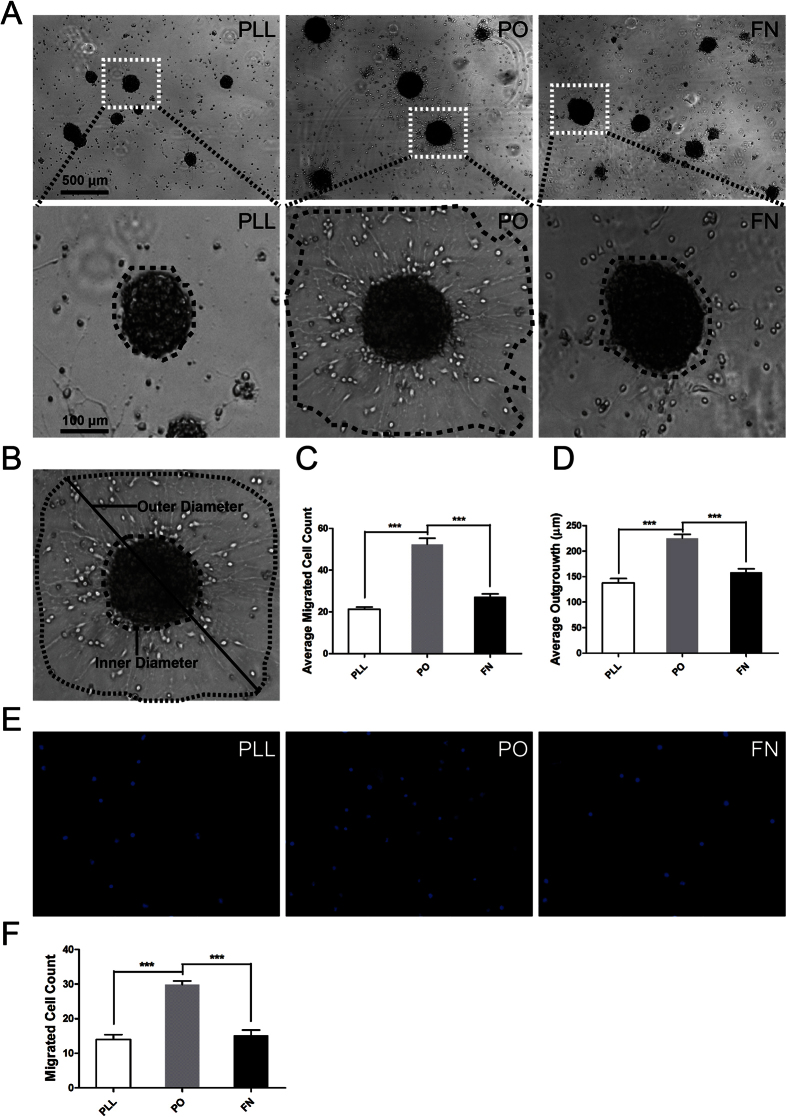
PO evidently enhanced migration of NSPCs. **(A)** Neurospheres were plated in different substrates pre-coated 24-well plates and images were captured by phase contrast microscopy after 12 hours. Insets were magnified images from each photograph at low magnification. **(B)** NPSCs migration was determined by the longest outer diameter of neurosphere migration normalized to inner sphere diameter and reported in micron. **(C)** Summarized graph showed the number of migration cells from neurospheres on PLL, PO and FN. ***P < 0.01, one-way ANOVA followed by Tukey’s post hoc test (n = 6 for each group)**. (D)** Quantitative analysis of migration distance from neurospheres on PLL, PO and FN respectively. ***P < 0.01, one-way ANOVA followed by Tukey’s post hoc test (n = 6 for each group). **(E)** Transwell assays to evaluated the migration potential of NSPCs on PLL, PO and FN. **(F)** Summarized graph indicated the number of migration cells from upper chambers to lower ones pre-coated with PLL, PO and FN. ***P < 0.01, one-way ANOVA followed by Tukey’s post hoc test (pooled data from five independent experiments). The data are presented as the mean ± SEM.

**Figure 3:**
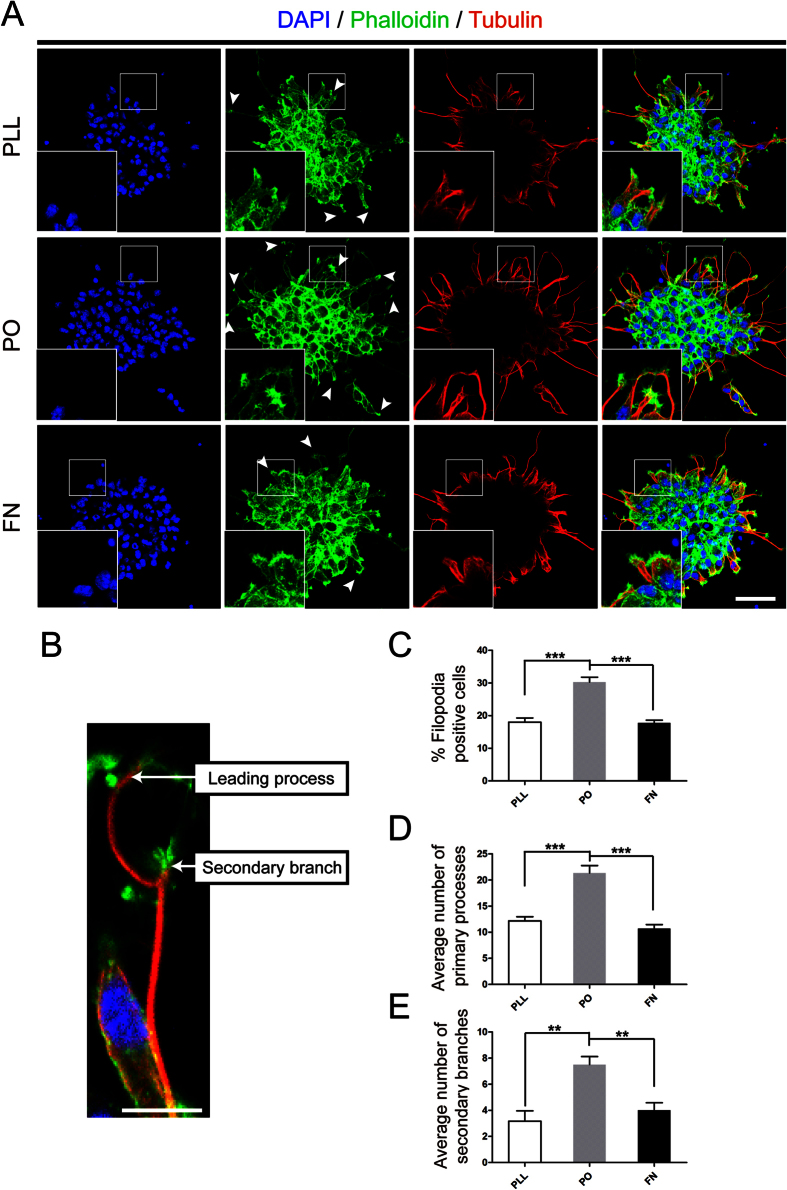
PO promoted filopodia formation. **(A)** Phalloidin staining (green) assessed the nubmer of filopodia formation and tubulin immunostaining (red) images showed the cage-like microtubule structure in neurospheres growing on PLL, PO and FN. Cell nuclei were stained with DAPI in blue. Arrows indicated the standard filopodia formation in each group. Scale bar: 20 μm. **(B)** An example of phalloidin and tubulin-labeled NSPCs with a secondary branch and a leading process. Scale bar: 20 μm. **(C)** Bar graph demonstrated the percent of filopodia formation on PLL, PO and FN. ***P < 0.01, one-way ANOVA followed by Tukey’s post hoc test (n = 6 for each group). **(D)** Quantitative analysis of average number of primary leading processes on PLL, PO and FN respectively. ***P < 0.01, one-way ANOVA followed by Tukey’s post hoc test (n = 6 for each group). **(E)** Quantitative analysis of average number of secondary branches on PLL, PO and FN respectively. **P < 0.05, one-way ANOVA followed by Tukey’s post hoc test (n = 6 for each group).

We have previously shown there are no obviously different effect on death or survival of NSPCs among PLL, PO and FN^9^. In the present study, we used the same adherent culture system to investigate their effects on migration of NSPCs. NSPCs were plated on different substrates in the enrichment medium with 20 ng/ml bFGF and 20 ng/ml EGF according to the well-known standard method^19^. First, the number and distance of migrated cells were evaluated under phase contrast microscopy. Results indicated that NPSCs on PO migrated from neurospheres were significant increase than that on PLL and FN, both in average migrated cell number and distance (Fig. 2A–D). Next, transwell assays were used to confirm the results collected from previous test. The data demonstrated that Millipore Transwell inserts pre-treated with PO could greatly increase cell mobility, compared with PLL and FN (Fig. 2E,F). Together, these data suggested that PO bear- the capacity to enhance the migration of NSPCs. To understand why PO enhanced the migration of NSPCs, we performed phalloidin staining to assess the filopodia formation, an indicator of cell polarization at the beginning of migration^20^. Meanwhile, we stained the expression of tubulin, a symbol of cage-like microtubule structure^21^, to evaluate the morphological structure changes in NSPCs. The results indicated that NSPCs on PO had larger percent of filopodia formation (Fig. 3A,C). Moreover, The average number of processes of NSPCs on PO was sharper increase than that on PLL and FN, including leading processes (Fig. 3A,D) and secondary branches (Fig. 3A,E). While, there were no obvious differentce in cage-like microtubule structure (Fig. 3A). These data demonstrated that PO promoted migration of NSPCs through facilitating filopodia formation.

### ACTN4 expressed in NSPCs and involved in promoting migration of NSPCs on PO

ACTN4 directly mediates cell migration and filopodia formation in non-muscle cells^17^. The above results had indicated that PO could enhance the formation of actin filaments. Meanwhile, NSPCs are non-muscle cells. Therefore, we hypothesize that ACTN4 involve in promoting migration of NSPCs.

**Figure 4:**
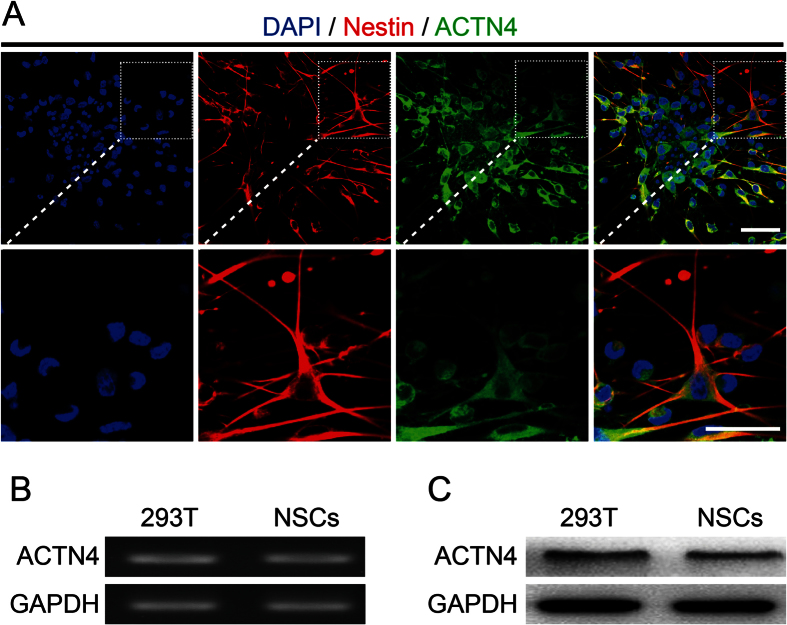
ACTN4 expressed in NSPCs. **(A)** The immunostaining images indicated the co-labeled of the Nestin (red) and ACTN4 (green) of neurospheres after seeded on PO for 12 hours. Insets were magnified images from each photograph at low magnification. Cell nuclei were stained with DAPI in blue. Scale bar: 20 μm. **(B)** RT-PCR assays demonstrated the mRNA expression of ACTN4 in NSPCs, with 293 T cell line as positive control. **(C)** Western blotting assays indicated the protein expression of ACTN4 in NSPCs, with 293 T cell line as positive control.

**Figure 5:**
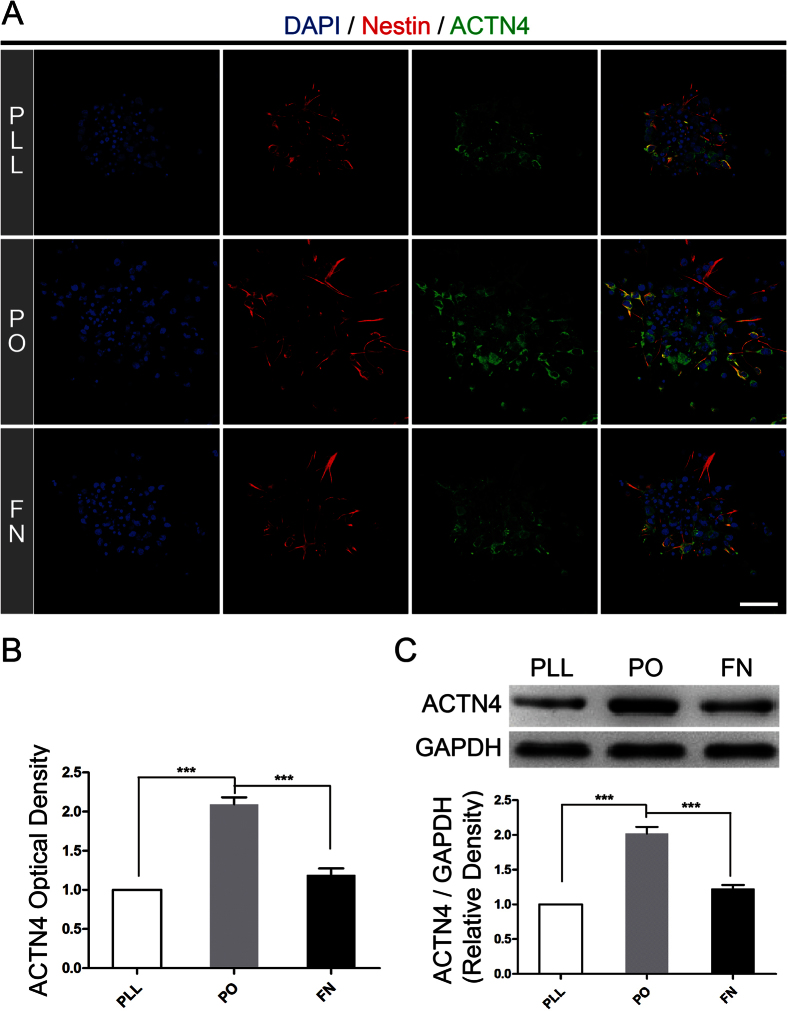
PO upregulated ACTN4 expression, compared to PLL and FN. **(A)** The immunostaining images indicated ACTN4 expression (green) on PLL, PO and FN. Cell nuclei were stained with DAPI in blue. Scale bar: 20 μm. **(B)** Bar graphs summarized semi-quantitative results from (**A**). ***P < 0.01, one-way ANOVA followed by Tukey’s post hoc test (n = 3 for each group). **(C)** Western blotting assays demonstrated the different expression level of ACTN4 on PLL, PO and FN after 12 hours for migration. Bands were analyzed using the Image Lab™ software for relative density and normalized to GAPDH control. ***P < 0.01, one-way ANOVA followed by Tukey’s post hoc test (n = 3 for each group).

To certify our hypothesis, we firstly identified ACTN4 expressed in NSPCs by immunofluorescence (Fig. 4A). Next, RT-PCR and western blotting assays were used to determine the mRNA and protein expression of ACTN4 in NSPCs, with 293T cell line as positive control to assure the results obtained from immunofluorescence respectively. The data from RT-PCR assays showed that ACTN4 mRNA expressed in NSPCs was almost the same level, compared to the positive control (Fig. 4B). Meanwhile, the western blotting assays indicated the same tendency as mRNA expression (Fig. 4C). Furthermore, immunostaining images showed that NSPCs expressed the highest level of ACTN4, compared with PLL and FN (Fig. 5A,B). In addition, western blotting assays were used to determine the expression level of ACTN4 among PLL, PO and FN. The results illustrated that NSPCs grown on PO expressed the highest level of ACTN4 (Fig. 5C), which confirmed the data obtained from immunofluorescence. In general, these data indicated that ACTN4 expressed in NSPCs and involved in PO induced migration of NSPCs through promoting filopodia formation. As far as we knew, it was the first time to identify the expression of ACTN4 and its function in NSPCs.

### ACTN4 played a pivotal role in migration induced by PO

**Figure 6:**
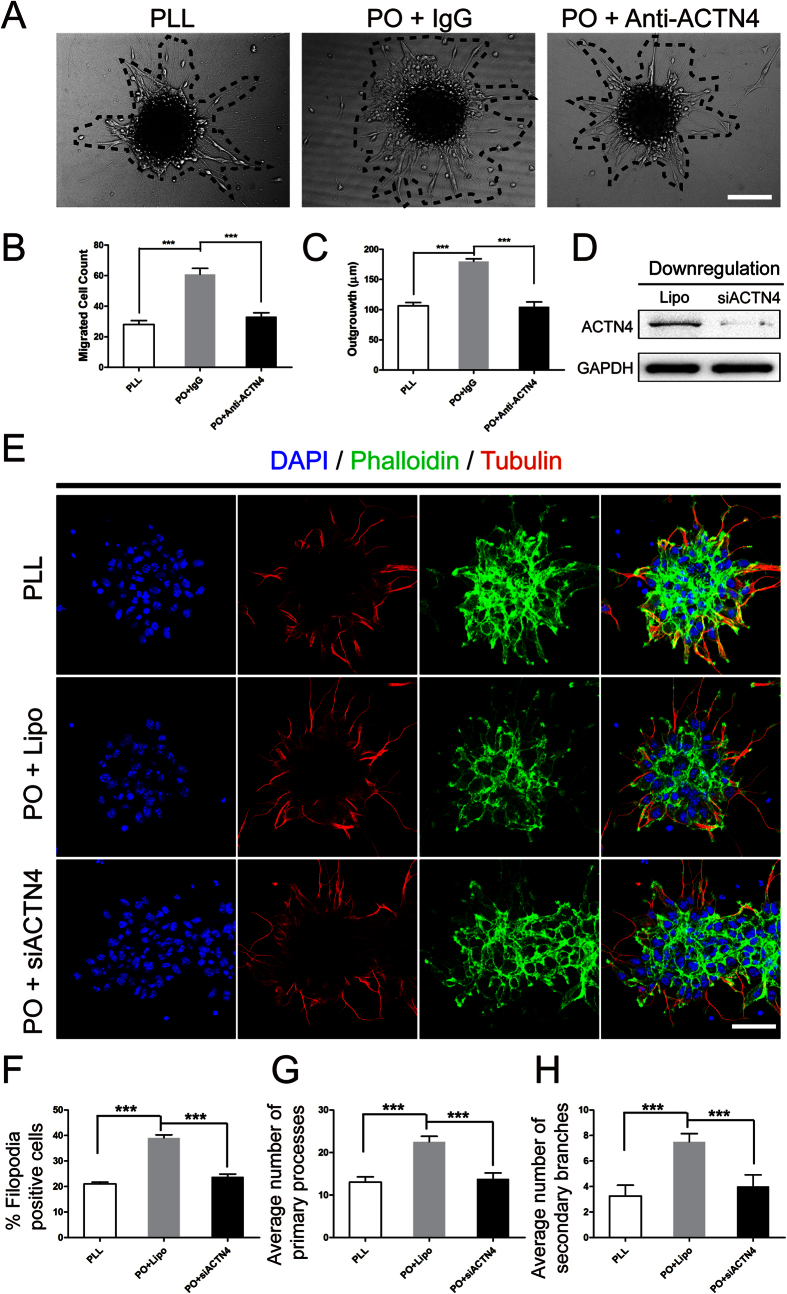
ACTN4 played a pivotal role in migration induced by PO. (**A**) Neutralizing Antibody Assays showed the role of ACTN4 playing in migration induced by PO. Neurospheres were plated in PLL or PO pre-coated 24-well plates and images were captured by phase contrast microscopy after 12 hours. Normal rabbit IgG was used as a negative control. (**B**) Summarized graph indicated the number of migration cells from neurospheres after neutralizing antibody assays, with PLL as control. ***P < 0.01, one-way ANOVA followed by Tukey’s post hoc test (n = 5 for each group). (**C**) Quantitative analysis of migration distance from neurospheres after neutralizing antibody assays, with PLL as control. ***P < 0.01, one-way ANOVA followed by Tukey’s post hoc test (n = 5 for each group). (**D**) Blotting bands showed downregulation of ACTN4 using siRNA transfection. Lipofectamine (Lipo) was used as a negative control. (**E**) The effect of ACTN4 on filopodia formation using phalloidin staining (green) and the cage-like microtubule structure with tubulin immunostaining (red) in neurospheres with/without ACTN4 downregulation using siRNA transfection, compared to PLL control. (**F**) Bar graph indicated the percent of filopodia formation. ***P < 0.01, one-way ANOVA followed by Tukey’s post hoc test (n = 5 for each group). (**G**) Quantitative analysis of average number of primary leading processes. ***P < 0.01, one-way ANOVA followed by Tukey’s post hoc test (n = 5 for each group). (**H**) Quantitative analysis of average number of secondary branches. ***P < 0.01, one-way ANOVA followed by Tukey’s post hoc test (n = 5 for each group).

To further unravel the role of ACTN4 playing in PO induced migration of NSPCs, we first used either 1 μg/ml neutralizing antibody against rabbit ACTN4 or control IgG diluted in 1 ml enrichment medium on PO, meanwhile, PLL as control. The data indicated that NSPCs cultured on PO migrated larger number than that on PLL. While, the migrated cell number and distance decreased significantly with the addition of neutralizing antibody after incubation for 12 hours (Fig. 6A–C). Furthmore, to confirm the results above, the downregulation of ACTN4 expression by siRNA were performed. The results indicated the percent of filopodia formation decreased greatly to the PLL level (Fig. 6D,E). At the same time, the migrated cell number and distance showed the same tendency as the treatment of neutralizing antibody (Fig. 6D,F,G). Taken together, these data indicated that ACTN4 played a pivotal role in enhancing migration of NSPCs induced by PO and the underlying mechnism was promoting filopodia formation.

## Discussion

NSPCs, with the ability of multipotency and differentiation into three main subtypes of neural cells, hold promise for cell replacement therapy of many neurological disorders such as Parkinson’s disease^22^, traumatic brain injury^23^ and ischemic stroke^24^,^25^,^26^. To date, how to obtain sufficient NSPCs and direct their proper differentiation is a big challenge. Our previous study has indicated that PO could significantly increase NSPCs proliferation and induce preferred differentiation into neurons and oligodendrocytes *in vitro*^9^. While, study has shown that NSPCs proliferate in the Subventricular Zone (SVZ), but only a few proliferated NSPCs could migrate to the lesions after ischemic stroke in adult rats^10^. It implies that the shortage of functional NSPCs in lesions might due to the limitation of NSPCs migration potential after brain injury. Biomaterials for cell replacement therapies should facilitate proliferation, differentiation and migration of NSPCs. Our previous study has uncovered the effect of PO on proliferation and differentiation of NSPCs. Meanwhile, studies have proved that PLL is likely to enhance inflammatory responses^27^,^28^,^29^. And, FN would influence the proliferation potential of NSPCs *in vitro*^30^. While, PO usually serves as one component of alginate microcapsule system to deliver medicine and transplanted cells^31^,^32^. Hence, it is necessary to find out whether PO could improve the migratory potential of NSPCs and its underlying mechnism might be different from our previous study.

In the present study, we identify ACTN4 expressed in NSPCs and it plays a pivotal role in migration of NSPCs induced by PO. ACTN4, first cloned by Honda *et al.*^33^, acts as a significant mediator to regulate cytoskeletal organization of the actin network by cross-linking F-actin^17^,^34^. Previous studies have shown that increased ACTN4 expression promotes the motility of various cancer cell migration and metastasis^14^,^15^,^17^,^35^,^36^, while its downregulation evidently decreases the migration potential of lung cancer metastasis to the brain^36^. Here, our results indicate that ACTN4 facilitates NSPCs migration induced by PO, which was consistent with previous studies in malignant phenotypes of cancer cells^35^,^36^,^37^, compared to PLL and FN. It is the first time to provide exact evidence, to our limited knowledge, to illustrate the expression and its function of ACTN4 in NSPCs.

Meanwhile, PO enhances filopodia formation of NSPCs, which exhibits positive correlation with ACTN4 expression. ACTN4, one of actin-bundling proteins, could directly cross-link the filopodia formation^20^,^38^. Filopodia-thin, finger-like and highly dynamic actin-rich membrane protrusions, could extend out from cell edge when migration and its extension could be mediated by various molecules such as small GTPases of the Rho family (i.e. Rac1 and Cdc42)^33^,^39^. The NSPCs on PLL, PO and FN show different migratory potential due to the different expression levels of ACTN4 to regulate filopodia formation. As we previously showed that NSPCs on PO increased higher level of p-ERK^9^, which is a regulator to mediate the expression of Rac1 and RhoA^40^,^41^. Hence, the underlying mechnism for PO promoting migration of NSPCs might be due to the activition of ERK/small GTPases/ACTN4 signaling cascade. But which member of small GTPases is dominated needs to be addressed in the future investigation.

In general, our present study has revealed pivotal effect of PO on NSPCs migration and its possible underlying mechanism, which is a significant supplement of our previous study. Together, our results suggest that PO could serve as a better candidate for cultivating NSPCs *in vitro*, even for transplantation biomaterial *in vivo*, in comparison with PLL and FN, for basic and preclinical research after various neurological diseases and injuries.

## Materials and Methods

### Animal

This study was carried out according to the China’s animal welfare legislation for the care and use of animals and approved by the Third Military Medical University Chongqing, China. Every effort was made to minimize the number of animals and relieve their suffering. E14.5 Sprague-Dawley rats were sacrificed after anesthetized with pentobarbital (60 mg/kg intraperitoneally).

### Primary Neural Stem/Progenitor Cells Culture

Primary rat NSPCs from the cortices of fetal E14.5 Sprague–Dawley pups were isolated and cultured as previously described^9^. Neocortical tissues were trypsinized by 0.25% trypsin (GIBCO, Grand Island, NY) for 30 minutes at 37 °C. Soybean trypsin inhibitor (2.8 mg/mL, Roche Diagnosis, Mannheim, Germany) was in addition to inhibit trypsin activity. After washing twice with DMEM/F12 medium (GIBCO, Grand Island, NY), the samples were triturated using a fire-polished Pasteur pipette and passed through a 100 μm Nylon cell strainer (BD Falcon, San Jose, CA) to harvest dissociated cell suspensions. Then, they were seeded at an initial cell density of 1 × 10^5^ cells/mL in DMEM/F12 medium supplemented with 20 ng/ml recombinant murine FGF-basic (Peprotech, Rocky Hill, NJ), 20 ng/ml recombinant murine EGF (Peprotech, Rocky Hill, NJ) and 2% B-27 supplement (GIBCO, Grand Island, NY) for 24 hours at 37 °C under humidified 5% CO_2_ conditions. Neurospheres were seeded on 35-mm different pre-coated dishes (18 hours) after washing twice with sterile cell culture grade water according to the manufacturer’s instructions respectively. The pre-coated substrates were as follows: 100 μg/ml Poly-L-lysine (Sigma-Aldrich, St. Louis, MO), 10 μg/ml poly-L-ornithine (Sigma-Aldrich, St. Louis, MO) and 20 μg/ml Fibronectin (Sigma-Aldrich, St. Louis, MO). Half volume of culture medium was exchanged every 3 days.

### Immunofluorescence

For fluorescence, neurospheres, adhered to different pre-coated coverslips, were fixed with 4% paraformaldehyde in 0.01 M phosphate-buffered saline (pH 7.4) for 2 hours at room temperature and blocked with 5% v/v fetal bovine serum or with 0.5% v/v Triton-X 100 (Sigma-Aldrich, St. Louis, MO) in PBS. Neurospheres were incubated with rabbit polyclonal to Doublecortin (DCX, 1:200, Abcam, Cambridge, UK), rabbit polyclonal to glial fibrillary acidic protein (GFAP, 1:100, Proteintech Group, Inc, Beijing, China), rabbit polyclonal to Olig2 (1:100, Proteintech Group, Inc, Beijing, China), rabbit polyclonal to ACTN4 (1:100, Proteintech Group, Inc, Beijing, China), mouse monoclonal to Tubulin (1:100, Beyotime, Beijing, China) or mouse monoclonal to Nestin (1:200, Abcam, Cambridge, UK) overnight at 4 °C and then relative fluorescence secondary antibodies were incubated for 2 hours at room temperature. Cell nuclei were stained with 4′-6-Diamidino-2-phenylindole (DAPI, Sigma-Aldrich, St. Louis, MO) for 10 minutes at room temperature. Then, coverslips were mounted onto glass slides and the images were captured by confocal microscope (Carl Zeiss, LSM780, Weimar, German) and examined using Zen 2011 software (Carl Zeiss, Weimar, Germany).

### NPSCs Migration Assay

For neurospheres migration assay, neurospheres were plated on different substrates pre-coated 24-well plates and the quantification methods were previously described^42^. Images were captured by phase contrast microscopy at 10 × once every 2 h for 1 day allowing for the tracking of NPSCs migration out of the neurospheres. Phase contrast images (*n* = 6 per sample well) were analyzed for longest sphere diameter as shown in Fig. 2B using a custom-designed MatLab program (MathWorks, Inc., Natick, MA) and were normalized to baseline measurements taken 2 h after plating. All neurosphere experiments were performed in four wells, and results were representative of at least three independent experiments.

For single cell migration assays, NSPCs were allowed to migrate through 12 μm pore Millicell cell culture inserts (Millipore, Temecula, CA) in accordance with the manufacturer’s instructions as previously^43^. Insert membranes were coated with substrates as described previously and NPSCs were plated in mitogenic growth-factor free media (n = 5 replicates per group). The upper chambers were seeded with 100 μl (1 × 10^5^) NSPCs. The lower chambers were filled with 600 μl DMEM/F-12 medium supplemented with 10% FBS which served as a chemoattractant. The NSPCs were allowed to migrate from the upper to lower chambers for 12 hours at 37 °C in a humidified incubator with 5% CO_2_. Non-migratory cells were removed from the top of the membrane with a cotton swab and the cells attached to the lower surface of membrane were fixed in 4% paraformaldehyde at room temperature for 30 min and counterstained with DAPI, and the number was counted under fluorescence microscope. A total of 5 fields were counted for each transwell filter.

### Western Blotting

Neurospheres adhered to different precoated dishes were homogenized with RIPA (Sigma-Aldrich, St. Louis, MO) supplemented with protease inhibitor cocktail (Roche, Indianapolia, IN, USA). The protein concentration was measured by enhanced BCA Protein Assay Kit (Beyotime, Beijing, China). Proteins (15 μg/lane) were separated by 10% SDS-PAGE under reducing conditions and electroblotted to polyvinylidene difluoride membranes (Roche, Indianapolia, IN, USA). After membranes were blocked in 5% skimmed milk in TBST at room temperature for 2 hours. Then, they were incubated with rabbit polyclonal to ACTN4 (1:1000, Proteintech Group, Inc, Beijing, China) and mouse monoclonal to GAPDH (1:1000, Santa Cruz Biotechnology, CA, USA) overnight at 4 °C. After incubation with peroxidase-conjugated (HRP)-conjugated secondary IgG (Zsgb-bio, 1:5000) for 2 hours at room temprature. All membranes were detected by ChemiDoc™ XRS^+^ imaging system (Bio-Rad, California, USA) using the WesternBright ECL Kits (Advansta, Menlo Park, CA, USA). Densitometric measurement of each membrane was performed using Image Lab™ software (Bio-Rad, California, USA). GAPDH, an internal control, was used to normalize the expression level of each protein.

### Reverse Transcription Polymerase Chain Reaction

Total RNA was extracted from neurospheres with TaKaRa MiniBEST Universal RNA Extraction Kit according to the manufacturer’s instructions (TaKaRa Bio Inc. Tokyo, Japan) and contaminating DNA was depleted with RNase-free DNase (Qiagen, Valencia, CA). Total RNA (2 μg) was reverse transcribed into cDNA with PrimeScript™ II 1st Strand cDNA Synthesis Kit (TaKaRa Bio Inc. Tokyo, Japan) and an aliquot of cDNA mixture (0.2%) was used as polymerase chain reaction (PCR) templates. ACTN4 primers were purchased from Invitrogen (rs138139611, Thermo Fisher Scientific, Waltham, MA, USA), GAPDH (forward, 5′-GGC CCC TCT GGA AAG CTG TG-3′; reverse, 5′-CCA GGC GGC ATG GCA GAT C-3′). The annealing temperature for PCR was 55 °C and carried out for 30 cycles. Gels were imaged by ChemiDoc™ XRS^+^ System (Bio-Rad, California, USA).

### Filopodia Detection

To visualize F-actin for filopodia formation assays, cells were incubated with 488-phalloidin (Life Technologies, Waltham, MA, USA). Samples were mounted in the ProLong Gold Antifade Reagent with DAPI (Sigma-Aldrich, St. Louis, MO), and results were analyzed with a confocal microscope (Carl Zeiss, LSM780, Weimar, German) and examined using Zen 2011 software (Carl Zeiss, Weimar, Germany).

### Neutralizing Antibody Assay

For neutralizing antibody group, neurospheres were incubated in 1 μg/ml neutralizing antibody against rabbit ACTN4 antibody and normal rabbit IgG as control before seeding in PO pre-coated 24-well plates. Then neurospheres were plated in 24-well plates and images were captured by phase contrast microscopy at 10 × once every 2 h for 1 day allowing for the tracking of NPSCs migration out of the neurosphere. The quantification methods were as previously described.

### ACTN4 siRNA transfection

ACTN4-specific siRNA (sc-43102) was purchased from Santa Cruz Biotechnology (CA, USA). Transfection of siRNA was performed using Lipofectamine RNAiMAX (Lipo, Invitrogen, Waltham, MA, USA) transfection reagent according to the manufacturer’s instructions. And the same amout of Lipofectamine RNAiMAX was used as negative control.

### Statistical methods

All data were showed as mean ± SEM and statistical analyses were carried out with SPSS v19.0 (SPSS Inc, Chicago, IL). One-way ANOVA followed by Tukey’s post hoc test was used to define statistical significance. p < 0.05 was considered statistically significant and p < 0.01 was considered as obvious statistical significance.

## Additional Information

**How to cite this article**: Ge, H. *et al.* Poly-L-ornithine enhances migration of neural stem/progenitor cells via promoting α-Actinin 4 binding to actin filaments. *Sci. Rep.* **6**, 37681; doi: 10.1038/srep37681 (2016).

**Publisher’s note:** Springer Nature remains neutral with regard to jurisdictional claims in published maps and institutional affiliations.
